# Cost of anterior *versus* posterior spinal instrumented fusion for thoracolumbar (Lenke V/VI) adolescent idiopathic scoliosis

**DOI:** 10.1007/s43390-025-01272-z

**Published:** 2026-01-21

**Authors:** Alekos A. Theologis, Monty Khela, Mohammad Diab

**Affiliations:** 1 Department of Orthopædic Surgery, University of California - San Francisco (UCSF), San Francisco, CA USA; 2 School of Medicine, Creighton University, Omaha, NE USA

**Keywords:** Adolescent idiopathic scoliosis, Thoracolumbar, Anterior spinal instrumented fusion, Posterior spinal instrumented fusion, Direct costs, Intensive care unit, Length of stay

## Abstract

**Purpose:**

To compare direct costs associated with anterior spinal instrumented fusions (ASIF) and posterior spinal instrumented fusions (PSIF) for thoracolumbar adolescent idiopathic scoliosis (AIS).

**Methods:**

A retrospective analysis was conducted of adolescents (ages 10–18 years) who underwent ASIF or PSIF for thoracolumbar AIS by a single surgeon. Demographics, clinical and surgical details, and inpatient post-operative outcomes were analyzed. Direct costs were obtained from medical billing data, including supplies, instrumentation, operating room services, room and board, and ICU admissions.

**Results:**

17 patients (13 girls; average age 15.3 ± 1.9 years) met inclusion criteria and were analyzed. There were no significant differences in age, major thoracolumbar Cobb angle, or thoracic Cobb angle between groups. PSIF had significantly more instrumented levels (6 ± 0.9 *v.* 5 levels; p < 0.01) and shorter operative times (308 ± 25.6 min vs. 447 ± 39.2 min; *p* < 0.01). ASIF incurred higher total costs ($72,174 ± $19,550 *v.* $66,552 ± $14,019; p = 0.04) and direct costs ($40,161 ± $3,668 *v.* $34,469 ± $7,846; *p* = 0.04), largely due to more ICU admissions and greater hospital lengths of stay (LOS). Excluding 2 ASIF patients with prolonged hospitalizations, direct costs between ASIF ($39,990 ± $3,855) and PSIF ($34,469 ± $7,846) were not statistically different (*p* > 0.05).

**Conclusions:**

While ASIF had significantly greater direct costs due to more postoperative ICU admissions and longer hospital LOS, exclusion of outliers resulted in similar average direct costs between approaches. ASIF saved one treated level on average. Number of implants, operating room services, ICU admissions and LOS are the principal drivers of cost.

## Introduction

Adolescent idiopathic scoliosis (AIS) is a three-dimensional twisting of the spine that leads to disfigurement early and functional impairment late. Half of AIS curves are thoracic; by contrast, slightly more than 10% are thoracolumbar/lumbar [1]. The principal goals of surgical management of AIS are to arrest curve progression by achieving a durable fusion, preserve or restore spinal balance, enhance appearance by correction and ultimately improve quality of life. There is uncertainty about superiority of surgical treatment of isolated thoracolumbar/lumbar curves by anterior spinal instrumented fusion (ASIF) *versus* posterior spinal instrumented fusion (PSIF).

Trends in spinal instrumented fusions in AIS show a decline in the anterior approach in favor of the posterior approach [2]. The principal advantage of the anterior approach is the power of pushing the spine to correct deformity, which enables the saving of fusion levels. The increasing burden of junctional kyphosis has renewed interest in the anterior approach, which does not devitalize the paraspinous muscles. Proponents of the posterior approach have questioned the saving of levels given newer operative techniques, in particular posterior column osteotomies, and instrumentation, in particular all pedicle screw constructs. Furthermore, the posterior approach is extensile (from occiput to sacrum), utilitarian (every curve type), familiar (*e.g.* does not require an access surgeon) and quicker. We investigate cost as one more factor that may aid decision making between anterior or posterior approach for thoracolumbar spinal instrumented fusions to treat thoracolumbar AIS.

## Methods

### Study design and patient population

**Fig. 1:**
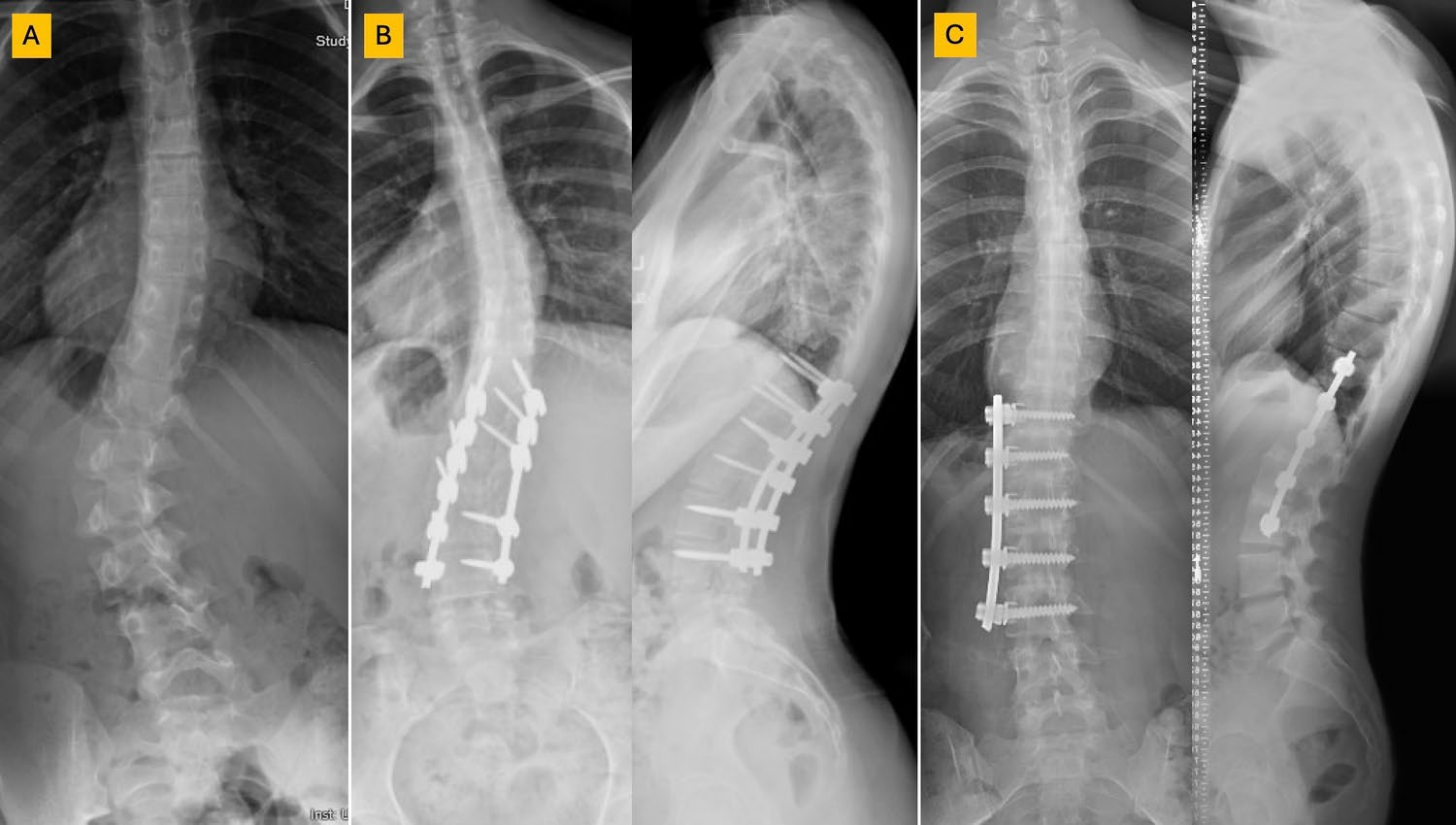
Radiographic examples of adolescent idiopathic thoracolumbar scoliosis (**A**; Lenke Type V) treated with anterior instrumented fusion (**B**) or posterior instrumented fusion (**C**)

**Fig. 2:**
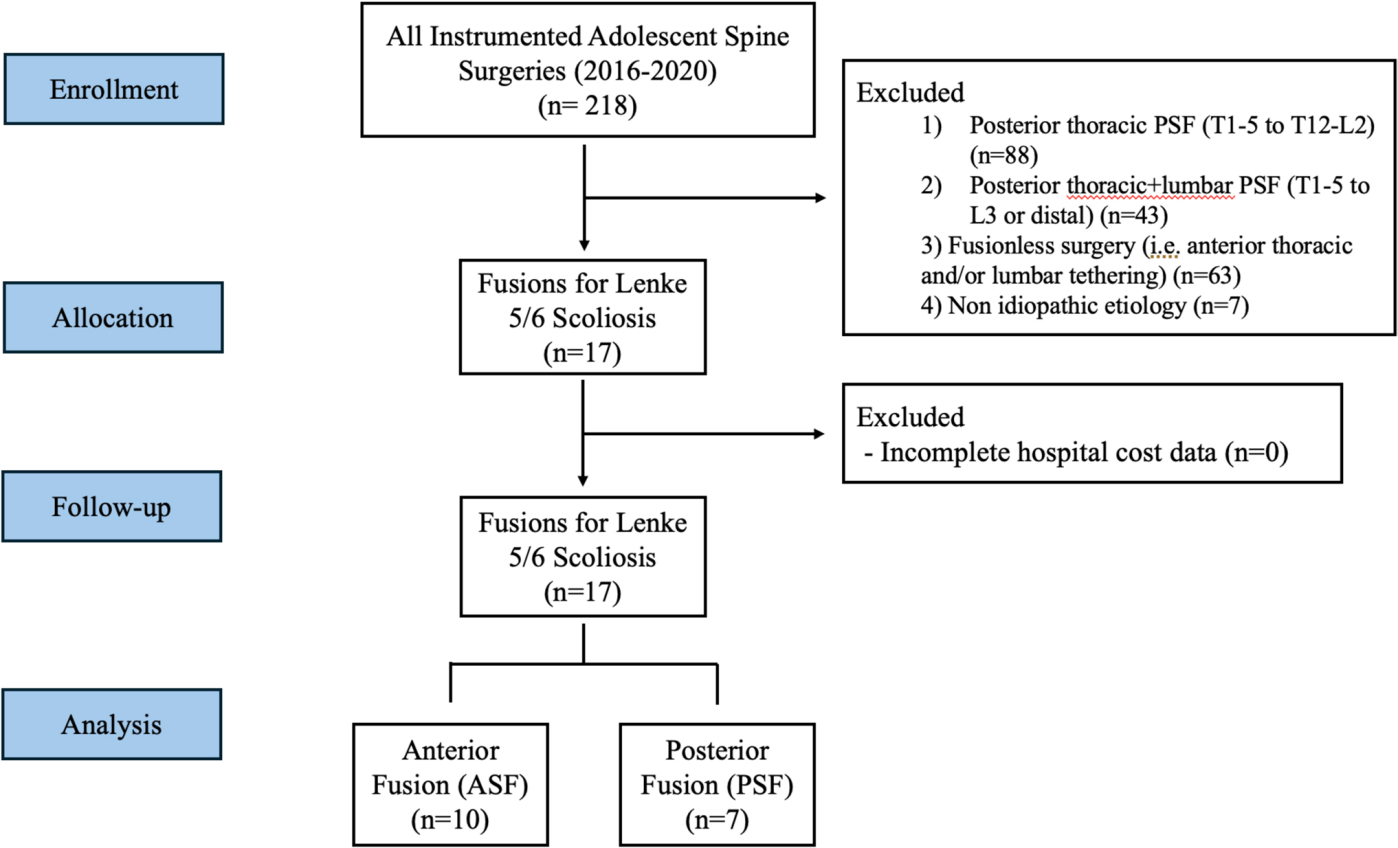
CONSORT flow chart of included patients. * PSF = posterior spinal instrumented fusions; ASF = anterior spinal instrumented fusions

This study was approved by our Institutional Review Board approval. A retrospective review was performed of all adolescents (age 10–18 years) with AIS who underwent primary spinal instrumentation between January 2016 and December 2020 at a single academic medical center in the United States of America by a single surgeon. Patients were included for analysis if they had an instrumented fusion of only a major lumbar/thoracolumbar curve (Fig. 1). Secondary operation(s), non-idiopathic diagnosis, fusionless operations (i.e. vertebral body tethering), and incomplete data were exclusion criteria (Fig. 2).

Clinical and direct cost data, as outlined below, were collected for the cohort and compared between patients based upon approach: anterior *versus* posterior (Fig. 1). All anterior approaches were performed by the senior surgeon of this study, including access, closure and postoperative management. Instrumentation for anterior and posterior approaches was the same [Universal Spine System (USS)™; Depuy Synthes].

### Data collection

Data were collected from the electronic medical record, including demographics [age, sex, body mass index (BMI)], operative measures [curve magnitude, laterality, operative time, estimated blood loss (EBL), number of levels fused, implant construct], and post-operative outcomes [hospital length of stay (LOS), ICU admissions, complications].

Direct costs (the expense incurred to deliver health care services) (rather than charges – the amount asked by providers for a health care good or service, which would appear on a medical bill) were calculated for index operations from medical billing reports generated and validated by the hospital cost accounting team at our institution. Financial data included all facility costs, divided into 4 categories: surgical supplies (implants and other surgical supplies), services [including inpatient services such as physical therapy (PT)/occupational therapy (OT) and intraoperative services such as operating room staffing fees, neuromonitoring, fluoroscopy], room and care, and pharmacy. While total costs are reported as the summation of direct and indirect costs, specific subcategorization of indirect costs (such as hospital administrative and facilities overhead, health information management, accounting and billing, housekeeping), surgeon fees, and costs for skilled nursing facility, acute rehabilitation, and revision operations are not able to be provided, as they are not made available to us from our institution. Additionally, surgeon fees were excluded because surgeons’ fees represent a direct cost to payers but do not represent the direct cost of providing care for a specific patient. Anesthesia fees were considered a component of the operating room costs. Operative time included total time the patient was in the operating room rather than time of incision to closure.

### Statistical analysis

Descriptive statistics were used to summarize the patient demographics, surgical details, and cost components. Continuous variables were presented as means and standard deviations, while categorical variables were presented as frequencies and percentages. Comparisons between ASIF and PSIF groups were made using independent Student’s *t*-tests for continuous variables and chi-square tests for categorical variables.

## Results

### Patient demographics and operative details (Table 1)

**Table 1:** Comparative patient demographics, radiographic deformity parameters, operative details, and hospital length of stay

	All	Anterior	Posterior	*p*
N	17	10	7	n/a
Age (years)	15.3 ± 1.9	15.4 ± 1.8	15.0 ± 2.1	0.31
(Avg ± SD; range)	(11.7—18)	(12.8—18)	(11.7 – 17.6)	
Gender				
Male	4	2	2	
Female	13	8	5	
Major Thoracolumbar Cobb (^0^)	51.2 ± 6.4	50.5 ± 4.6	52.3 ± 8.6	0.29
(Avg ± SD; range)	(45—65)	(45—60)	(45—65)	
Compensatory Thoracic Cobb (^0^)	28.3 ± 5.0	27.7 ± 5.4	29.1 ± 4.6	0.29
(Avg ± SD; range)	(20—38)	(20—38)	(22—36)	
Thoracolumbar Curve Apex				
Left	14	8	6	
Right	3	2	1	
# Levels Fused/Instrumented	5.4 ± 0.7	5.0 ± 0	6.0 ± 0.9	< 0.01
(Avg ± SD; range)	(5—7)	(5)	(5—7)	
Operative Time (mins)	391 ± 79	447 ± 39.2	308 ± 25.6	< 0.01
(Avg ± SD; range)	(283—528)	(389—528)	(283—348)	
Hospital Length of Stay (day)	4.9 ± 1.6	5.0 ± 2.1	4.0 ± 0.5	0.15
(Avg ± SD; range)	(4—10)	(4—10)	(4—5)	

17 adolescent patients (mean age 15.3 ± 1.9 years, range 11.7–18 years) met inclusion criteria. There were 13 girls and 4 boys. The anterior approach was performed on 10 patients, while the posterior approach was performed on 7 patients. There was no significant difference in the ages of patients between the anterior and posterior groups (*p* = 0.31). Major thoracolumbar/lumbar Cobb angle (ASIF: 51.2^0^ ± 6.4^0^ *v.* PSIF: 52.3^0^ ± 8.6^0^) and thoracic Cobb angle (ASIF: 27.7^0^ ± 5.4^0^ *v.* PSIF: 29.1^0^ ± 4.6^0^) were statistically similar between the two groups (p < 0.05). The majority of curves had a left apex (ASIF: 80%; PSIF: 85.7%; *p* > 0.05). PSIF was associated with more levels fused/instrumented 6 ± 0.9 levels (range: 5–7), compared with ASIF, which consistently involved 5 levels (*p* < 0.01). PSIF had shorter operative times (308 ± 25.6 min; range 283—348 min) compared with ASIF (447 ± 39.2 min; range 389 to 528 min) (*p* < 0.01). After operation, ASIF had more ICU admissions and longer hospital LOS (2 patients > 7 days).

### Direct Cost data (Tables 2 & 3)

**Table 2:** Comparative total and direct cost data for children who underwent anterior and posterior instrumented fusion for thoracolumbar adolescent idiopathic scoliosis

	All	Anterior	Posterior	*P*
N	17	10	7	n/a
Total Costs ($)	66,099 ± 18,857	72,174 ± 19,550	56,552 ± 14,019	**0.04**
	(42,876 – 127,002)	(57,163 – 127,002)	(42,876 – 83,560)	
Total Direct Costs ($)	37,818 ± 6,244	40,161 ± 3,668	34,469 ± 7,846	**0.04**
	(26,565 – 49,090)	(34,138 – 45,425)	(26,565 – 49,090)	
Direct Costs – Subcategories ($)				
Anesthesia	1,156 ± 428	1,241 ± 450	873 ± 178	0.10
	(761 – 2,352)	(908 – 2,352)	(761 – 1,079)	
Lab	439 ± 216	433 ± 228	458 ± 212	0.43
	(127—813)	(127—813)	(311—702)	
Supplies	14,841 ± 4,334	14,680 ± 4,556	15,378 ± 4,328	0.41
	(5,042 – 20,363)	(5,042 – 18,639)	(12,568 – 20,363)	
OT/PT	853 ± 288	796 ± 292	1,025 ± 233	0.13
	(483 – 1,358)	(483 – 1,358)	(795 – 1,261)	
OR Services	10,389 ± 3,798	10,256 ± 3,680	10,834 ± 5,024	0.41
	(6,658 – 19,867)	(6,658 – 19,867)	(7,761 – 16,632)	
Pharmacy	602 ± 277	662 ± 242	400 ± 340	0.08
	(69 – 1,052)	(351 – 1,052)	(69—749)	
Radiology	1,435 ± 450	1,485 ± 373	1,273 ± 730	0.25
	(702 – 2,248)	(1,050 – 2,248)	(702 – 2,096)	
PACU	1,133 ± 710	1,263 ± 758	1,163 ± 226	0.42
	(0 – 2,730)	(598 – 2,730)	(933 – 1,385)	
Room/board	6,493 ± 2,053	6,367 ± 2,298	6,912 ± 1,089	0.35
	(4,194 – 11,527)	(4,194 – 11,527)	(5,787 – 7,960)	
ICU	4,966 ± 2,065	5,511 ± 2,149	3,331.9	n/a
	(3,052 – 7,026)	(3,052 – 7,026)		
Other	1,707 ± 1,126	1,850 ± 1,255	1,229 ± 280	0.21
	(511 – 4,499)	(511 – 4,499)	(913 – 1,446)	

**Table 3:** Details of 2 outlier children with extended length of stay (> 7 days) following anterior spinal instrumented fusions for thoracolumbar AIS

	#1	#3
Age (years)	13.5	18.4
Sex	Girl	Girl
Thoracolumbar curve magnitude (^0^)	50	49
Thoracolumbar curve apex	Left	Right
Operation	T11-L3 anterior instrumented fusion	T11-L3 anterior instrumented fusion
Operative time (mins)	462	389
Hospital LOS (days)	10	8
Reason for extended LOS	C. Diff infection	Difficult pain control
Cost Data		
Total costs ($)	70,993	61,812
Direct costs ($)	43,665	38,025

^*^LOS = length of stay

Data for direct costs are presented in Table 2. Total costs were greater for ASIF ($72,174 ± $19,550; range: $57,163-$127,002) compared with PSIF ($66,552 ± $14,019; range: $42,876-$83,560) (*p* = 0.04), as were direct costs for AISF ($40,161 ± $3,668; range: $34,138-$45,425) compared with PSIF ($34,469 ± $7,846; range: $26,565-$49,090) (*p* = 0.04). The four major drivers of direct costs for both approaches were anesthesia (ASIF: $1,241 ± $450; PSIF: $873 ± $178; *p* = 0.10), pharmacy (ASIF: $662 ± $242; PSIF: $400 ± $340; *p* = 0.08), radiology (ASIF: $1,485 ± $373; PSIF: $1,272.6 ± $730; *p* = 0.25), and ICU admissions [ASIF: $5,511 ± $2,149; PSIF: $3,331.9 (n = 1); *p* = n/a].

Two patients who underwent anterior operations had a hospital LOS greater than 7 days due to *Clostridium difficile* infection and difficult pain control (Table 3). When these 2 patients were excluded from the cost analysis, the direct costs for ASIF and PSIF were not statistically different (anterior: $39,990 ± $ 3,855; posterior: $34,469 ± $7,846; *p* > 0.05).

## Discussion

From 1997 to 2012, the cost of scoliosis surgery increased dramatically, with hospital charges tripling from approximately $55,000 to over $175,000 [3, 4]. This increase is multifactorial, including surgical techniques such as posterior column osteotomies, higher facility costs, and the use of more (*e.g.* anchoring every level) and more complex instrumentation.

The choice between ASIF and PSIF for treating thoracolumbar/lumbar AIS requires a comprehensive, individualized approach considering various factors. Factors that may be taken into account include severity and flexibility of the curve, spinal motion preservation (saving a level), risk of revision operation, and patient/parent preferences. By contrast with adults, abdominal wall morbidity has not been identified as significant in adolescents. The economic impact of each surgical option has not previously been considered.

Surgical management of AIS is ever-evolving. An analysis of the National Inpatient Sample from 1998 to 2011 showed a preference for posterior approaches over anterior approaches for thoracic curves, but equipoise for lumbar curves [2]. The latter finding has its origins in the potential saving of levels, and thereby increasing residual motion, by “pushing” the deformity from the front of the spine with screws rather than “pulling” on the deformity from the back with early hook constructs. Our study as well as others show that this remains valid today with pedicle screws [5–8]. In our study, all posterior constructs included L4 as the lowest instrumented vertebrae, while all anterior operations stopped at L3. Saving a fusion level (L3-4), and thereby reducing loss of spine motion in the lumbar spine, is difficult to quantify in the short-term, but may be more evident in the future. As > 50% of lumbar lordosis arises from below L3, stopping at L3 allows one additional segment of motion through which adolescents and young adults may compensate their sagittal profiles. As the lumbar spine stiffens and loses lordosis with increasing age, the need to compensate through open motion segments to maintain an upright posture becomes more important. In turn, having 3 (compared to 2) distal motion segments available for compensation lends credence to the notion that there is considerable value in preserving as much motion as possible in adolescent patients.

There are other advantages of ASIF compared with PSIF. Infection after anterior spine surgery is exceedingly rare compared with posterior [9]. ASIF achieves excellent correction of trunk rotation and radiographic outcomes, particularly for thoracolumbar curves [10, 11]. Like infection, junctional kyphosis does not seem to be the problem for ASIF that it is after PSIF due to preservation of the paraspinous muscles [12]; again like infection, repair of junctional kyphosis is a significant cost burden [13].

The anterior approach is technically more challenging and has a unique complication profile. While our senior surgeon performs his own approaches, many institutions enlist a separate access surgeon, which adds to cost. The anterior approach involves retroperitoneal dissection and release of the diaphragm, which risks pulmonary complications (i.e. pleural effusion, pneumothorax, prolonged chest tube placement), vascular injury, sympathetic chain dysfunction, and other visceral disturbances (bowel injury, ureteral injury, chylothorax) that may result in increased intensive care unit (ICU) stays, longer hospitalizations, and higher costs [5, 14–19]. Compared with the posterior approach, the anterior approach is less extensile. The posterior approach has been reported to be associated with shorter hospital stays and fewer complications compared to ASIF, which appeared to be reasons for the lower associated costs in our study [2, 6, 8, 10, 12].

In our study, we identified that the major drivers of cost for both approaches to be instrumentation, OR services, room/board, and ICU admissions. ASIF had significantly higher total and direct costs compared with PSIF, principally due to ICU admissions and longer LOS. However, costs between ASIF and PSIF were statistically similar when 2 patients with extended LOS (> 7 days) due to *Clostridium difficile* and difficult pain control were excluded from the analysis. In addition, our practice of admitting anterior patients to the ICU for the first night after operation is based upon the concept of maintaining spinal cord perfusion with pressure support as needed in the event of sacrifice of a potential artery of Adamkiewicz during the approach [20]. We acknowledge that this is not a universal practice, further approximating the costs of the two approaches.

The results of this study need to be considered in the context of its limitations. The retrospective nature of this study introduces potential bias and jeopardizes comprehensive evaluation of all factors that may influence the evaluated outcome measures. Additionally, this study only includes patients from a single-center in the United States of America, which may limit its external validity to other institutions or patient populations. However, the fact that one surgeon performed all operations with the same instrumentation system reduces potential variability in costs between approaches. A further limitation is the relatively small sample size. While other potentially perceived limitations of the study are the absence of health-related quality of life outcome scores (not the customary practice of the primary surgeon to administer to patients), exclusion of postoperative radiographic alignment parameters, rates of pseudarthroses, these all have been focuses of numerous previous investigations and are not felt to contribute to perioperative costs, which is the primary focus of this investigation. Despite these limitations, our findings provide insights that can inform surgical practices and improve patient care in AIS treatment. While cost may not be a significant differentiating factor in the choice of anterior *versus* posterior approach for the surgical treatment of thoracolumbar/lumbar AIS in our study, data from multiple centers and multiple surgeons throughout the entire country are needed to gain a broader appreciation for the differential costs between anterior and posterior approaches for thoracolumbar/lumbar AIS instrumented fusions. Ultimately, long-term quality-adjusted life year (QALY) data that take into account revision operations (i.e. pseudarthrosis, junctional pathology, infections, etc.) will be instrumental in fully elucidating the relative cost effectiveness of each approach for this patient population.

## Conclusions

In conclusion, this study uniquely contributes insight into direct costs of ASIF and PSIF for thoracolumbar AIS. Specifically, major drivers of cost for both operations are instrumentation, OR services, room/board, and ICU admissions. While ASIF had significantly greater direct costs due to more ICU admissions post-op and more episodes of prolonged hospital LOS, exclusion of outliers resulted in similar average direct costs between ASIF and PSIF. In turn, minimizing LOS, ICU admissions, and postoperative complications are important to contain costs. Future multi-center studies will be important to validate our findings and guide decision-making around surgical care for AIS patients in a healthcare landscape that aims to provide value-based care.
